# Prevalence and determinants of common mental illness among adult residents of Harari Regional State, Eastern Ethiopia

**DOI:** 10.11604/pamj.2017.28.262.12508

**Published:** 2017-11-24

**Authors:** Gari Hunduma, Mulugeta Girma, Tesfaye Digaffe, Fitsum Weldegebreal, Assefa Tola

**Affiliations:** 1 Haramaya University, College of Health and Medical Sciences, School of Nursing and Midwifery, Harar, Ethiopia; 2 Haramaya University, College of Health and Medical Sciences, Department of Medical Laboratory Science, Harar, Ethiopia; 3 Harar Health Sciences College, Harar, Ethiopia

**Keywords:** Common mental illness, adult residents, harari regional state, Eastern Ethiopia

## Abstract

**Introduction:**

Common mental disorders include depression, anxiety and somatoform disorders are a public health problem in developed as well as developing countries. It represents a psychiatric morbidity with significant prevalence, affecting all stages of life and cause suffering to the individuals, their family and communities. Despite this fact, little information about the prevalence of common mental illness is available from low and middle-income countries including Ethiopia. The aim of this study was to determine the magnitude of common mental disorders and its associated factors among adult residents of Harari Region.

**Methods:**

Comparative cross-sectional, quantitative community-based survey was conducted From February 1, 2016 to March 30, 2016 in Harari Regional State using multi-stage sampling technique. A total of 968 residents was selected using two stage sampling technique. Of this 901 were participated in the study. Validated and Pretested Self reported questionnaire (SQR_20) was used to determine the maginitude of common mental disorders. Data was entered and analyzed using Epi-info version 3.5.1 and SPSS-17 for windows statistical packages. Univirate, Bi-variate and multivariate logistic regression analysis with 95% CI was employed in order to infer associations.

**Results:**

The prevalence of common mental illnesses among adults in our study area was 14.9%. The most common neurotic symptoms in this study were often head ache (23.2%), sleep badly (16%) and poor appetite (13.8%). Substance use like Khat chewing (48.2%), tobacco use (38.2%) and alcohol use (10.5%) was highly prevalent health problem among study participant. In multivariate logistic regression analysis, respondents age between 25-34 years, 35-44 years, 45-54 years and above 55years were 6.4 times (AOR 6.377; 95% CI: 2.280-17.835), 5.9 times (AOR 5.900; 95% CI: 2.243-14.859), 5.6 times (AOR 5.648; 95% CI: 2.200-14.50) and 4.1 times (AOR 4.110; 95% CI: 1.363-12.393) more likely having common mental illnesses than those age between 15-24 years, respectively. The occurrence of common mental illness was twice (AOR: 2.162; 95% CI 1.254-3.728) higher among respondents earn less than the average monthly income than those earn more than average monthly income. The odds of developing common mental illnesses were 6.6 times (AOR 6.653; 95% CI: 1.640-6.992) higher among adults with medically confirmed physical disability than those without physical disability. Similarly, adults who chewed Khat were 2.3 times (AOR 2.305; 95% CI: 1.484-3.579) more likely having common mental illnesses than those who did not chew Khat. Adults with emotional stress were twice (AOR 2.063; 95% CI: 1.176-3.619) higher chance to have common mental illnesses than adults without emotional stress.

**Conclusion:**

This study had reveals that common mental disorders are major public health problems. Advancing age, low average family monthly income, Khat chewing and emotional stress were independent predictors of common mental illnesses. Whereas sex, place of residence, educational status, marital status, occupation, family size, financial stress, taking alcohol, tobacco use and family history of mental illnesses were not statistically associated with common mental illnesses.

## Introduction

Mental illness is a public health problem in developed as well as developing countries [1]. Globally, neuropsychiatric conditions account for 9.8% burden of the diseases [2, 3]. Atleast one in four people are affected by amental health problem at some point in their lives [4]. Mental and behavioural disorders are found in every countries at all stages of life [5]. The burden of chronic non communicable disease is emerging as a major public health challenge worldwide, especially in developing countries where these diseases have been assumed to be less common [6]. Five of the ten leading causes of disability and premature death worldwide are related with psychiatric conditions. Globally, about 25% of the population will develop mental illness at some stage in their lives, and 12% to the global Burden of disease is from the low-income countries [7]. Depression is the third leading cause of disease burden worldwide; representing 4.3% of total disability adjusted life years and predicted to become the second leading cause of the global disease burden by the year 2020 [8]. In Ethiopia, mental health problems accounts for 12.45% of the burden of diseases, 12% of the people are suffering from mental health problems [9, 10]. The poor or inexistent mental health care, both in terms of the offer of services and development of policies on health protection and promotion is due to lack of information about the mental health status. Currently no available published study on community based common mental disorders in Harari Region. Therefore, the present researchers believe that this problem which affects the living condition of the society is really a gap that needs to be addressed. This study was, therefore, intended to determine the magnitude of common mental disorders and its associated factors among adult residents of Harari Region.

## Methods

**Study area**: The study was conducted in Harari People Regional State. The region is one of the nine regions in the country; it is located in the eastern part of Ethiopia. Harar, the capital city of the region, is located on a hill top to the eastern extension of the Ethiopian highlands, about 510Km away from Addis Ababa. Its altitudes range from 1800 to 2000 meters. The region situated on the area of 342.2 km^2^ in which the rural area constitutes 323.7 km^2^ while the urban area has about 19.1 km^2^ only. It is the smallest region in Ethiopia and surrounded by different districts of Oromia Region, namely Kombolcha and Jarso in Northern side, Gursum and Babile in Northeast, Fedis in south east and Haramaya in the West side respectively. Harar also lies within fertile coffee growing districts and agricultural fields, producing various products particularly chat, fruits/ vegetables, and several kinds of grains [11]. Based onMOH, Health and Health Related Indicator, the total population of the region is estimated to be 203,834 in 2003 EC and this makes Harari Region least populous region in the country. About 54% of the population lives in the urban area while the remaining 46% live in rural area. The people of Harari region earn their livelihood from trade, agriculture and employment by government and the private sector [12]. According to the currently adopted administrative structure, Harari Region is divided in to 19 kebeles (in urban area) and 17 peasant association (in rural areas). In the current system the district is responsible for management of Primary Health Care Unit (Health centre and Health post), while the management of Hospitals and Training Institution are under the RHB. The RHB is organized in to different departments and services, each with specific roles and responsibilities. At district level, there is one health coordinator and two experts. At the health post, level there is one nurse and two female HEWs. With regard to the number of facilities, the region has relatively a higher degree of Health Service Coverage (100%) as compared to the national level [11].

**Study design and period**: A community- based comparative cross-sectional survey was carried out among adults in five randomly selected woredas using multi-stage sampling technique. This study was conducted from February to March, 2016.

**Sample size determination and sampling procedure**: The sample size was determined by two proportion formula considering the study which was conducted in Oyo state of Nigeria [13]. The proportion of the psychiatric morbidities among urban and rural area were 18.4% and 28.4%, respectively.With a precision of 95% and the desired power of 80%. Including 15% loss to follow-up and design effect of 1.5, the final sample size for each of urban and rural area were 484 (a total of 968 households were included in the study). Multistage sampling technique was used to obtain a representative sample of the communities in Harari regional state as follows.

**Stage 1**: A sampling frame of all the woredas in Harari regional state was drawn and stratified into urban and rural areas. Two rural and three urban woredas were obtained by simple random sampling. According to this, Aboker, Amin Nur and Hakim woredas from urban and Erer and Sofi woredas from rural local government areas were selected.

**Stage 2:**: Sampling frame of all the kebeles in the selected woredas were drawn. The kebeles where the study carried out was randomly selected by simple random sample. The selected kebeles were kebele 02 and 03 from Amin woreda; kebele12 from Aboker woreda; kebele 18 from Hakim woreda; Hawaye from Erer woreda; and Awuberkele and Burka from Sofi woreda.

**Stage 3**: The total number of households in kebele 02 was 1084, in kebele 03 was 466, in kebele 12 was 1752, in kebele 18 was 1930, in Hawaye kebele was 1130 and in Awuberkele kebele was 1450 & Burka kebele was 1104. Based on proportionate allocation to size, the numbers of households selected in each kebele were determined. Accordingly, the number of houses selected were 188 in kebele 02; 80 in kebele 03; 303 in kebele 12; 333 in kebele 18; 139 in Hawaye kebele, 178 in Awuberkele kebele; and 135in Burka kebele.

**Stage 4**: Systematic sampling technique was employed to select the houses that were visited in the chosen communities. The sample fraction was 3 households in kebele 02, kebele 03, kebele 12 & kebele 18 and 4 households in Hawaye, Awuberkele & Burka kebeles. One resident aged 18 years and above selected by lottery methods and was interviewed in the households selected. A total of 968 household were recruited into the study.

**Data collection instrument:** The data were collected by using a self-reported questionnaire-20 (SRQ-20) which is developed by the World Health Organization (WHO) as a screening tool for common mental disorders. Originally (SRQ-20) it consist 25 questions; 20 related to neurotic symptoms, 4 concerning psychosis and 1 asking about convulsions. This study concentrates on the SRQ-20, which (consists of 20 yes/no questions) assesses presence of neurotic symptoms (anxiety, depression, psychosomatic). Mental illness was measured using the locally validated Self-Reported Questionnaire (score of ≥six indicating high levels of CMD). The SRQ has previously been translated into Amharic and validated in Ethiopia and it has been used for community surveys. It is reviewed to suit the local condition and translated to Amharic and afanoromo languages and then back to English to ensure its consistency. The survey questionnaire were pre-tested in two randomly selected kebeles (one from urban and one from rural) which was not be involved in the actual data collectionand the necessary modifications and correction was made to ensure its consistency. Using the questionnaire data were collected by twenty (20) trained HEW with experience in data collection and fluent speakers of afanoromo and Amharic languages. The interview was made by house-to-house visit in the presence of strong supervision.

**Data quality control**: To assure the data quality high emphasis was given in designing data collection instrument. The questionnaires were pre tested on 10% of the sample size in two randomly selected kebeles (one from urban and one from rural) which was not involved in the actual data collection to check consistency and length of time each questioners took, sampling method and techniques, as well as the skill of data collectors two weeks prior to the main data collection time. Training was provided for data collectors and supervisors on the objective of the study, the source of bias, method of data collection. Before data collection the questioners were checked its simplicity, clarity and understandability. Checking and re-checking of the data were employed to identify whether the data were completely filled or not by double data entry. Daily supervision of data collection process was implemented. To assess the consistency, 25% of the collected data were checked in a daily based.

**Data analysis**: First the collected data were entered in to Epi Info version 3.5.1 then exported to SPSS ver.20 software package. The entered data were cleaned, edited, coded and recoded. Bi-variate and multivariate logistic regression analysis with 95% confidence interval were employed in order to infer associations and predictions. Initially, each variable was entered into a logistic regression model to determine presence of statistical significant association between independent variables and the dependent variable. Logistic regression model was used to identify independently associated factors. All independent variables that associated with the dependent variable in bivariate analyses with a P-value of < 0.05 were included in the final logistic model.

**Ethical considerations**: Ethical approval and clearance was obtained from the Haramaya University, College of Health and Medical Science IHRERC. An official letter of co-operation was written to Harari regional health bureaus in addition to personal communications by the investigators. The objective and purpose of the study was informed to the sample population in order to give genuine information, Based on the written and signed informed consent participants were informed that they have the right to withdraw or refuse to participate in the study at any time. A letter explaining the need for and benefit of the study, the method of questing, confidentiality, privacy and others were attached to the cover page of the questioner. A person with characteristics of mental illness defined as a cut-off point greater than or equal to 6 during SRQ-20 screening wich then approved by investegators and had not on treatment were immediately communicated to the psychiatric clinic in order to facilitate the way the person obtain farther investigation and proper management by his/her own expense. If in case the participant can't pay the medical cost investigators cover the cost of treatment.

## Results

**Socio demographic characteristics of respondents**: A total of 901 adults were participated in this study that make response rate 93.1%. Out of the total number of respondents 558 (61.9%) were male. The age of the respondents was ranged from 18 years to 83 years with mean (+SD) of 34.04 + 9.31 year. The majority of participants 475(52.7%) were age between 25-34 years followed by age 15-24years which accounted 20.9%. Four hundred fifty six (50.6%) were rural, the rest were urban dwellers. Regarding their marital status, about two third (68.3%) of participants were married. Most of the respondents were Muslim (80.4%) by their religion followed by orthodox Christian (17.6%). Educational level of the respondents comprises from unable to read and write to 12 plus being dominated by primary school 270 (30%) and secondary school and 241 (26.7%). About 2/3(67.4%) of the respondents were Oromo by ethnicity, followed by Amhara (16.0%) and Harari (6.2%). Occupationally, 378 (42%) were farmer, 167(18.5%) were Government employees and 123(13.7%) were merchants. The respondent's average monthly income was ranging from zero to 3000 Ethiopian birr with median and mean (+SD) monthly income of 600 (IQR 742.2) and 831.89 + 643.22 Birr respectively. About one-third (33.6%) of respondents did have monthly income between 401-800 birr & 28.6% did have monthly income less than 400 Ethiopian birr. The family size of respondents was ranging from 1 to 12 with mean (+SD) of 3.96 + 2.13. Majority 482 (53.5%) of respondents were living within family of 3-5 members followed by respondents of 1-2 family size which accounted 26.3% (Table 1).

**Table 1:** Socio-demographic characteristics of respondents’ of common mental illnesses among adult residents of Harari region, Eastern Ethiopia, March, 2016

Variables		Frequency	Percent
Sex	Male	558	61.9%
	Female	343	38.1%
	15-24	188	20.9%
Age	25-34	475	52.7%
	35-44	164	18.2%
	45-54	47	5.2%
	55+	27	3.0%
Residence	Urban	445	49.4%
	Rural	456	50.6%
Marital status	Single	149	16.5%
	Married	615	68.3%
	Divorced	55	6.1%
	Widowed	68	7.5%
	Separated	14	1.6%
Religion	Muslim	724	80.4%
	Orthodox	159	17.6%
	Other	18	2.0%
Educational status	Unable to read and write	185	20.5%
	Able to read and write only	138	15.3%
	Primary(grade 1-8)	270	30.0%
	Secondary(grade 9-12)	241	26.7%
	12 + grade	67	7.4%
Ethnicity	Oromo	607	67.4%
	Amhara	144	16.0%
	Harari	56	6.2%
	Somali	29	3.2%
	Gurage	41	4.6%
	Other	24	2.6%
Average Monthly income	<400 birr	258	28.6%
	401-800 birr	303	33.6%
	801-1500 birr	181	20.1%
	>1501 birr	159	17.6%
Occupational status	Farmer	378	42.0%
	Merchant	123	13.7%
	Gov't Employed	167	18.5%
	private employed	56	6.2%
	Student	62	6.9%
	Daily laborer	105	11.7%
	Other	10	1.1%
Family size	1-2	237	26.3%
	3-5	482	53.5%
	6+	182	20.2%

**Magnitude of common mental illnesses**: A locally validated self-reported questionnaire-20 (SRQ-20) was used as screening tool to assess presence of common mental disorders among respondents. This SRQ-20 consists of 20 yes/no questions which can assess of neurotic symptoms (anxiety, depression, psychosomatic). Each yes/no response of each respondent was first summing up. Finally those respondents scored more than or equal to six were categorized as having common Mental illness and those scored less than 6 categorized as free from common mental illness. The respondents' score of those neurotic symptoms were ranged from 0 to 20. More than half (57.9%) of the respondents were reported that they had no any of those symptoms while 11 (1.2%) reported they had all of neurotic symptoms. In this study 134 participants were respond as having ≥ six neurotic symptoms, that make the prevalence of common mental illnesses 14.9% (Table 2). The most common neurotic symptoms in this study were often head ache (23.2%), sleep badly (16%) and poor appetite (13.8%). In contrary, the least complained symptoms were uncomfortable feeling in the stomach (7.8%), easily freighted (10%) and having shaking hands (10%) (Table 3).

**Table 2:** Prevalence of common mental disorders among adult living in Harari regional state, Eastern Ethiopia, March, 2016

Common Mental illness status	Frequency	Percent
Have common mental illness	134	14.9
No common mental illness	767	85.1
Total	901	100.0
**Score of SRQ-20**		
None (0)	522	57.9
1-5.9	245	27.2
6-20	134	14.9
Total	901	100.0

**Table 3:** Self-reported neurotic symptoms distribution of adults living in Harari region state, Eastern Ethiopia, March, 2016

Self-reported neurotic symptoms		Frequency	Percent
Often have head ache	Yes	209	23.2
	No	692	76.8
Have poor appetite	Yes	124	13.8
	No	777	86.2
Sleep badly	Yes	144	16.0
	No	757	84.0
Easily frightened	Yes	90	10.0
	No	811	90.0
Have shaking hands	Yes	90	10.0
	No	811	90.0
Feel nervous, tens or worried	Yes	116	12.9
	No	785	87.1
Have poor digestion	Yes	116	12.9
	No	785	87.1
Have trouble thinking clearly	Yes	102	11.3
	No	799	88.7
Being unhappy	Yes	114	12.7
	No	787	87.3
Cry more than usual	Yes	103	11.4
	No	798	88.6
Find difficult to enjoy your daily activities	Yes	105	11.7
	No	796	88.3
Find difficult in decision making in day to day life	Yes	106	11.8
	No	795	88.2
Daily work suffering	Yes	107	11.9
	No	794	88.1
Unable to play a useful part in life	Yes	106	11.8
	No	795	88.2
Lost interest in things	Yes	105	11.7
	No	796	88.3
Feeling as worthless person	Yes	94	10.4
	No	807	89.6
Thought of ending your life been on your mind	Yes	93	10.3
	No	808	89.7
Feeling tired all the time	Yes	110	12.2
	No	791	87.8
Uncomfortable feelings in your stomach	Yes	70	7.8
	No	831	92.2
Easily tired	Yes	103	11.4
	No	798	88.6

### Factors associated with common mental illnesses

**Occurrence of stressful life events and substance use**: Out of 901 participants of this study, 132 (14.7%) had history of death of someone close to their family, 106 (11.8%) experienced legal issues and 99 (11.0%) were separated from their spouses. Death of someone close to respondent was occurred more commonly among adults with common mental illnesses (11.9%) than adults without common mental illnesses (5.7%). From those stressful events, the least reported events by the respondents were being violated by other person 5(0.6%), having severely sick, physically abuse or disability close relative (3.2%), loss of job (3.3%). Five (3.7%) of adults with common mental illnesses and 16 (2.1%) of adults without common mental illnesses had family history of mental illnesses. Similarly 21 (15.7%) of adults with common mental illnesses and 68 (8.9%) of adults without common mental illnesses had emotional stress (Table 4). Almost half (48.2%) of respondents were chewing Khat in the last 3 months. In the other ward, around two-third (64.2%) of adults with common mental illnesses and 45.4% of adults without common mental illnesses were chewed khats in the last 3 months. Similarly 46.3% of adults with common mental illnesses and 36.8% of adults without common mental illnesses were smoke cigarette in the last 3 months that make prevalence of tobacco use 38.2%. About 8.2% & 11% of adults with common mental illnesses and without common mental illnesses had history of alcohol taking in the last 3 months respectively (Table 4).

**Table 4:** Stressful life event and substance use history among adults living in Harari region state, Eastern Ethiopia, March, 2016

EVENTS WITHIN THE LAST 6 MONTHS		Common mental disorder		Total Frequency (%)
		Yes Frequency (%)	No Frequency (%)	
Experience of sever sickness, physical abuse, or disability	Yes	12(9.0%)	64(8.3%)	76(8.4%)
	No	122(91.0%)	703(91.7%)	825(91.6%)
Death of someone close (father/mother, child) to the respondent	Yes	16 (11.9%)	44 (5.7%)	60 (6.7%)
	No	118(88.1%)	723 (94.3%)	841 (93.3%)
Severely sick, physical abuse, or disability of someone close to respondent	Yes	5(3.7%)	24(3.1%)	29 (3.2%)
	No	129 (96.3%)	743 (96.9%)	872 (96.8%)
Death of someone close to respondent’s family	Yes	21 (15.7%)	111(14.5%)	132 (14.7%)
	No	113(84.3%)	656 (85.5%)	769 (85.3%)
Experiencing separation from the spouse	Yes	22 (16.4%)	77 (10.0%)	99 (11.0%)
	No	112 (83.6%)	690 (90.0%)	802 (89.0%)
Experiencing loss of strong relationship or friend-ship	Yes	6 (4.5%)	28(3.7%)	34 (3.8%)
	No	128 (95.5%)	739(96.3%)	867 (96.2%)
Experiencing big problem with close friends	Yes	13 (9.7%)	70 (9.1%)	83 (9.2%)
	No	121(90.3%)	697 (90.9%)	818 (90.8%)
Experiencing big problem due to lack of money	Yes	16 (11.9%)	70 (9.1%)	86 (9.5%)
	No	118 (88.1%)	697 (90.9%)	815 (90.5%)
Lost of valuable property	Yes	5 (3.7%)	29 (3.8%)	34 (3.8%)
	No	129 (96.3%)	738 (96.2%)	867 (96.2%)
Experiencing any legal issues	Yes	20(14.9%)	86(11.2%)	106(11.8%)
	No	114(85.1%)	681(88.8%)	795(88.2%)
Lost of job	Yes	5 (3.7%)	25 (3.3%)	30 (3.3%)
	No	129 (96.3%)	742 (96.7%)	871 (96.7%)
Violated by other person	Yes	2 (1.5%)	3 (0.4%)	5 (0.6%)
	No	132 (98.5%)	764 (99.6%)	896 (99.4%)
Emotional stress	Yes	21(15.7%)	68(8.9%)	89(9.9%)
	No	113(84.3%)	699 (91.1%)	812(90.1%)
Financial stress	Yes	17(12.7%)	80(10.4%)	97(10.8%)
	No	117(87.3%)	687(89.6%)	804(89.2%)
Family history of mental illness of time	Yes	5(3.7%)	16 (2.1%)	21 (2.3%)
	No	129 (96.3%)	751(97.9%)	880(97.7%)
Medically confirmed physical disability of any time	Yes	4(3.0%)	5 (0.7%)	9(1.0%)
	No	130 (97.0%)	762 (99.3%)	892(99.0%)
Taking tobacco for the last 3 months	Yes	62(46.3%)	282 (36.8%)	344 (38.2%)
	No	72 (53.7%)	485 (63.2%)	557 (61.8%)
Taking alcohol for the last 3 month	Yes	11 (8.2%)	84(11.0%)	95(10.5%)
	No	123 (91.8%)	683 (89.0%)	806 (89.5%)
Taking khat for the last 3 months	Yes	86(64.2%)	348(45.4%)	434(48.2%)
	No	48(35.8%)	419(54.6%)	467(51.8%)

**Factors associated with common mental illnesses**: In bivariate logistic regression analysis, common mental illnesses were significantly associated with advancing age, educational status and average monthly income of the respondents. In contrary, among socio-demographic variables sex, place of residence, marital status, occupational status and family size were not associated with common mental illnesses (Table 5). Similarly bivariate logistic regression analysis showed that among stressful life events; death of respondents close one, experiencing separation from spouse and emotional stress were significantly associated with common mental illnesses. In addition, substance uses like taking khat and tobacco in the last 3 months were also significantly associated with common mental illnesses. But common mental illnesses were not associated with alcohol use, financial stress, family history of mental illness, experiencing legal issues, being severely sick, loss of job or valuable property and experiencing big problem with close friends (Table 6). In multivariate logistic regression analysis, respondents age between 25-34 years, 35-44 years, 45-54 years and above 55 years were 6.4 times (AOR 6.377; 95% CI: 2.280-17.835), 5.9 times (AOR 5.900; 95% CI: 2.243-14.859), 5.6 times (AOR 5.648; 95% CI: 2.200? 14.50) and 4.1 times (AOR 4.110; 95% CI: 1.363-12.393) more likely having common mental illnesses than those age between 15- 24 years, respectively. The occurrence of common mental illness was twice (AOR: 2.162; 95% CI 1.254-3.728) higher among respondents earn less than the average monthly income than those earn more than average monthly income. The odds of developing common mental illnesses were 6.6 times (AOR 6.653; 95% CI: 1.640-26.992) higher among adults with medically confirmed physical disability than those without physical disability. Similarly, adults who chewed Khat were 2.3 times (AOR 2.305; 95% CI: 1.484? 3.579) more likely having common mental illnesses than those who did not chew Khat. Adults with emotional stress were twice (AOR 2.063; 95% CI: 1.176-3.619) higher chance to have common mental illnesses than adults without emotional stress. In contrary, multivariate analysis of this study showed that common mental illnesses were not association with educational status, death of respondents closed one, experiencing separation from the spouse and taking tobacco (Table 7).

**Table 5:** Bivariate analysis of association between common mental illnesses and socio demographic characteristics among adults living in Harari region state, Eastern Ethiopia, March, 2016

Variables		Common mental disorder		p value	Crude OR
		Yes Frequency (%)	No Frequency (%)		
Sex	Male	81(60.4%)	477(62.2%)	0.702	1.00
	Female	53(39.6%)	290(37.8%)		0.929(0.638 -1.353)
Age	15-24	24 (17.9%)	164(21.4%)	0.002	1.00
	25-34	66 (49.3%)	409 (53.3%)	0.000	5.467 (2.287 -13.069) *
	35-44	23 (17.2%)	141(18.4%)	0.000	4.958 (2.222 11.060)*
	45-54	9(6.7%)	38(5.0%)	0.000	4.904 (2.039 -11.798)*
	55+	12(9.0%)	15(2.0%)	0.023	3.378 (1.181 -9.660) *
Residence	Urban	59 (44.0%)	386(50.3%)	0.179	1.00
	Rural	75 (56.0%)	381(49.7%)		0.776 (0.537-1.123)
Marital status	Single	17(12.7%)	132(17.2%)	0.116	1.00
	Married	88(65.7%)	527(68.7%)	0.284	2.118 (0.537 -8.358)
	Divorced	9(6.7%)	46(6.0%)	0.458	1.633(0.447 -5.971)
	Widowed	17(12.7%)	51(6.6%)	0.656	1.394 (0.323 -6.020)
	Separated	3(2.2%)	11(1.4%	0.777	0.818 (0.204 -3.284)
Educational status	Unable to read and write	41(30.6%)	144(18.8%)	0.021	1.00
	Able to read & write only	13(9.7%)	125(16.3%)	0.003	2.738 (1.403 - 5.341)*
	Grade 1-8	40(29.9%)	230(30.0%)	0.045	1.637 (1.010 - 2.653)*
	Grade 9-11	32(23.9%)	209(27.2%)	0.017	1.860 (1.118፟ - 3.093)*
	12 + grade	8(6.0%)	59(7.7%)	0.075	2.100 (.929-4.748)
Occupational status	Farmer	65(48.5%)	313(40.8%)	0.300	1.00
	Merchant	14(10.4%)	109(14.2%)	0.127	1.617 (0.872 - 2.997)
	Gov't Employed	18(13.4%)	149(19.4%)	0.057	1.719 (0.985 - 3.001)
	private employed	8(6.0%)	48(6.3%)	0.588	1.246 (0.563 - 2.758)
	Student	8(6.0%)	54(7.0%)	0.402	1.402(0.637 -3.086)
	Daily laborer	18(13.4%)	87(11.3%)	0.990	1.004 (0.566 -1.781)
	Other	3(2.2%)	7(0.9%)	0.303	0.485 (0.122 -1.923)
Average Monthly income	<831.89 birr	297(72.4%)	472(61.5%)	0.017	1.639 (1.092 - 2.458)*
	> 831.89 birr	37(27.6%)	265 (38.5%)	0.841	1.00
Family size	1-2	34(25.4%)	203(26.5%)	0.812	1.00
	3-5	75(56.0%)	407(53.1%)	0.670	0.909(0.586 1.410)*
	6+	25(18.7%)	157(20.5%)	0.859	1.052 (.603 -1.836)

**Table 6:** Bivariate analysis of association between common mental illnesses and stressful life events and substance use among adults living in Harari region state, March, 2016

Events within the last 6 months		Common mental disorder		p value	Crude OR
		Yes Frequency (%)	No Frequency (%)		
Experience of sever sickness, physical abuse or disability	Yes	12(9.0%)	64(8.3%)		1.00
	No	122(91.0%)	703(91.7%)	0.814	1.080 (0.566-2.061)
Death of respondent father/mother, child	Yes	16 (11.9%)	44 (5.7%)		1.00
	No	118(88.1%)	723 (94.3%)	0.009	2.228 (1.217-4.078)*
Severely sick, physical abuse, or disability of someone close to respondent	Yes	5(3.7%)	24(3.1%)		1.00
	No	129 (96.3%)	743 (96.9%)	0.716	1.200 (0.450-3.202)
Death of someone close to respondent’s family	Yes	21 (15.7%)	111(14.5%)		1.00
	No	113(84.3%)	656 (85.5%)	0.717	1.098 (0.661-1.824)
Experiencing separation from the spouse	Yes	22 (16.4%)	77 (10.0%)		1.00
	No	112 (83.6%)	690 (90.0%)	0.031	1.760 (1.053-2.943)*
Experiencing loss of strong relationship or friend-ship	Yes	6 (4.5%)	28(3.7%)		1.00
	No	128 (95.5%)	739(96.3%)	0.644	1.237 (0.502-3.047)
Experiencing big problem with close friends	Yes	13 (9.7%)	70 (9.1%)		1.00
	No	121(90.3%)	697 (90.9%)	0.832	1.070 (0.574-1.994)
Experiencing big problem due to lack of money	Yes	16 (11.9%)	70 (9.1%)		1.00
	No	118 (88.1%)	697 (90.9%)	0.308	1.350 (0.758-2.404)
Loss of valuable property	Yes	5 (3.7%)	29 (3.8%)		1.00
	No	129 (96.3%)	738 (96.2%)	0.978	0.986 (0.375-2.595)
Experiencing any legal issues	Yes	20(14.9%)	86(11.2%)		1.00
	No	114(85.1%)	681(88.8%)	0.220	1.389 (0.821-2.349)
Loss of job	Yes	5 (3.7%)	25 (3.3%)		1.00
	No	129 (96.3%)	742 (96.7%)	0.779	1.150 (0.433-3.060)
Family history of mental illness of time	Yes	5(3.7%)	16 (2.1%)		1.00
	No	129 (96.3%)	751(97.9%)	0.251	1.819 (0.655-5.052)
Taking tobacco in the last 3 months	Yes	62(46.3%)	282 (36.8%)		1.00
	No	72 (53.7%)	485 (63.2%)	0.037	1.481 (1.023-2.144)*
Taking alcohol in the last 3 month	Yes	11 (8.2%)	84(11.0%)		1.00
	No	123 (91.8%)	683 (89.0%)	0.342	0.727 (0.377-1.403)
Taking khat in the last 3 months	Yes	86(64.2%)	348(45.4%)		1.00
	No	48(35.8%)	419(54.6%)	0.000	2.157(1.474-3.157)*
Emotional stress	Yes	21(15.7%)	68(8.9%)		1.00
	No	113(84.3%)	699 (91.1%)	0.016	1.910 (1.127-3.239)*
Financial stress	Yes	17(12.7%)	80(10.4%)		1.00
	No	117(87.3%)	687(89.6%)	0.438	0.801 (0.458-1.402)

**Table 7:** Multivariate logistic regression analysis of determinant factors for common mental illnesses among adults living in Harari region state, Eastern Ethiopia, March, 2016

Variables		Common mental disorder		P value	Adjusted Or
		Yes Frequency (%)	No Frequency (%)		
Age	15-24	24 (17.9%)	164(21.4%)	0.004	1.00
	25-34	66 (49.3%)	409 (53.3%)	0.000	6.377 (2.280-17.835)*
	35-44	23 (17.2%)	141(18.4%)	0.000	5.900 (2.343- 14.859*
	45-54	9(6.7%)	38(5.0%)	0.000	5.648 (2.200-14.500)*
	55+	12(9.0%)	15(2.0%)	0.012	4.110 (1.363-12.393)*
Educational status	Illiterate	41(30.6%)	144(18.8%)	0.725	1.00
	Read and write only	13(9.7%)	125(16.3%)	0.747	1.182 (0.427-3.270)
	Grade 1-8	40(29.9%)	230(30.0%)	0.322	1.729 (0.586-5.104)
	Grade 9-12	32(23.9%)	209(27.2%)	0.879	1.075 (0.424-2.724)
	12 + grade	8(6.0%)	59(7.7%)	0.980	1.011 (0.418-2.448 )
Average Monthly income	<831.89 birr	297(72.4%)	472(61.5%)	0.006	2.162 (1.254-3.728)*
	> 831.89 birr	37(27.6%)	265 (38.5%)		1.00
Death of respondent father/mother, child	Yes	16(11.9%)	44 (5.7%)	0.780	0.926 (0.538-1.593 )
	No	118(88.1%)	723 (94.3%)		1.00
Experience separation from the spouse	Yes	22 (16.4%)	77 (10.0%)	0.174	1.478 (0.841-2.598)
	No	112 (83.6%)	690 (90.0%)		1.00
Medically confirmed physical disability	Yes	4(3.0%)	5 (0.7%)	0.008	6.653 (1.640-26.992 )*
	No	130 (97.0%)	762 (99.3%)		1.00
Taking tobacco inthe last 3 months	Yes	62(46.3%)	282 (36.8%)	0.456	0.847 (0.548-1.310 )
	No	72 (53.7%)	485 (63.2%)		1.00
Taking khat inthe last 3 months	Yes	86(64.2%)	348(45.4%)	0.000	2.305 (1.484-3.579 )*
	No	48(35.8%)	419(54.6%)		1.00
Emotional stress	Yes	21(15.7%)	68(8.9%)	0.012	2.063 (1.176-3.619) *
	No	113(84.3%)	699 (91.1%)		1.00

## Discussion

Mental illness is a public health problem that causes suffering to the individuals, their family and communities in developed as well as developing countries [1, 14]. The global burden of disease report revealed that common mental disorders account for 9.8% of the global burden of diseases [2, 3]. Worldwide it is estimated that lifetime prevalence ranges from 12.2-48.6% and 12-month prevalence between 8.4 and 29.1% [5]. But the magnitude and risk factors of common mental disorders are varying among different population. The prevalence of common mental illnesses among adults in our study area was 14.9%. This finding was consistent with previous studies conducted in Kenya (11%) [15], Borena Southern Ethiopia (14.6%) [16] and Addis Ababa (11.7%) [17]. But our finding was lower than many of the previous studies that reported prevalence of CMI among study population 21.9% in Nigeria [13], 22.7% in Jimma Ethiopia [18], 24.6% in Britain [19], 25.5% in Chile, Santiago [20], 27-30% in south Africa [21, 22], 29.9% in Brazil [14] and 30.3% in Britain [21].

This difference might be due to difference in data collection tools (some of those studies use CIS-R or GHQ), or due to difference in target population (urban population, disadvantaged Population) or time elapse between two studies. Similarly our finding was lower than the study conducted in four post conflict communities in Algeria (60.5%), Cambodia (53.4%), Ethiopia (23.6%) and Palestine and (29.1%) using the composite international diagnostic interview [23]. This high prevalence of CMI in those communities might be explained by the effect of conflict before the study conducted. Since serious threats such as conflicts and disasters were among several determinant factors of common mental illnesses, it was expected higher prevalence of CMI among those post conflict communities [24]. In contrary, our finding was 3 to 6 times higher than studies conducted in two urban areas (2.3% in Ilala Ilala and 4.1% in Saba Saba) of Tanzania [15]. This difference could be due to difference in study population and data collection tool. In our study, study population was adult living in both urban and rural kebeles whereas study population of Tanzania study was adults living in two urban areas. Similarly data collection tool of our study was self-reporting Questionnaire (SRQ-20) whereas Clinical Interview Schedule Revised (CIS-R) was used in Tanzania study.

In current study, 81 (14.5%) male and 53 (15.45%) female had common mental illnesses but there was no statistical significant association between sex and common mental illnesses. This finding was consistent with study conducted in Kenya [15]. But a number of previous studies showed that being female associated with higher risk of CMI [15, 17, 18, 21, 25]. Similarly, 13.3% of urban and 16.64% of rural residences had common mental illnesses. But there was no association between place of residence and common mental illnesses. This finding of higher prevalence of common mental illnesses among rural adults than urban was also observed in Nigeria study (18.4% in the urban areas and 28.4% in the rural areas) [13]. In current study advancing age was associated with increased likelihood of developing or having common mental illnesses. This finding was similar with several previous studies conducted in Brazil [14], Kenya [15], in Addis Ababa [17] and Butajira [25]. Like study conducted in in Addis Ababa [17], marital status of respondents did not show statistically significant associated with common mental illnesses. But some previous studies reported that marital status had significantly association with common mental illnesses [15, 26].

This difference might be due to difference in study population, study design and data collection tools. The first study was cohort study conducted on Indianwomen aged 18 to 50 yearsusing RCIS. Unlike many previous studies, educational status and employment status or occupation of respondent did not show statistical significant association with common mental illnesses [13-15, 18, 20, 27, 28]. As stated by Scott and Glyn, financial strain is a main predictor of future psychiatric morbidity [29]. In our study average family income was strongly associated with common mental illnesses. Adults with low average family income had two times higher chance of having mental illnesses than adults earned more than average monthly income. This association was also reported by many of the previous studies conducted in Nigeria [13], England, Wales, and Scotland [20], Brazil [14], Indian [26], two urban areas of Tanzania [15] and Butajira district rural Ethiopia [25]. In our study it was observed that about half (48.2%) of adults chewed khat.

This is because Khat chewing is one of common habit practiced among peoples living in the study area. In the other word, 64.2% of adults with common mental illnesses and 45.4% of adults without common mental illnesses were chewing Khat in the last 3 months. This difference was statistically significant that those adults who chewed Khat had 2.3 times higher chance of having common mental illnesses than those who did not chew khat. But alcohol taking and tobacco use did not show statistical association with common mental illnesses in this study. In contrary to this, other studies conducted in Brazil [14] and Indian [26] reported statistical significant relationship between tobacco use and common mental illnesses. About 3% of adults with common mental illnesses and 0.7% of adults without common mental illnesses had medically confirmed physical disability. This difference in proportion physical disability among adults with and without common mental illnesses was statistically significant. Adults with physical disability were almost 7 times higher chance of having common mental illnesses than adults without physical disability. This finding was also consistent with previous studies [13-15, 24, 26].

## Conclusion

This study significant proportion (one out of seven) of adults in Harari Regional state had common mental illnesses. Substance use like Khat chewing (48.2%), tobacco use (38.2%) and alcohol use (10.5%) was highly prevalent health problem among study participant. Advancing age, low average family monthly income, Khat chewing, having medically confirmed physical disability and emotional stress were independent predictors of common mental illnesses. Whereas sex, place of residence, educational status, marital status, occupation, family size, financial stress, taking alcohol, tobacco use and family history of mental illnesses were not statistically associated with common mental illnesses. Based on our findings, we would like to foreward the following recommendation to all concerned bodies including Harari Regional Health Bureau, Haramaya University, Researchers, mass media and community at large. Massive health awareness, promotion and education programs should be conducted regularly to the community about common mental illnesses and their risk factors. The negative health effect of substance use should be addressed continuously to the community through mass media. Due attention should be given to mental health aspect of those individual with low monthly family income, advanced age and physical disability. Further large scale research using both qualitative and quantitative approach, analytical design and different data collection tools should be conducted.

### What is known about this topic

- Common mental disorders;
- The public health problems of commen mental illiness.

### What this study adds

- Prevalence of common mental illness among adult residents in the region;
- Determinants of Common Mental Illness among adult residents in the region;
- Occurrence of stressful life events and substance use in the region.

## Competing interests

The authors declare no competing interest.
